# Association of neutrophil-to-lymphocyte-platelet ratio with residual renal function in early-stage peritoneal dialysis patients

**DOI:** 10.3389/fneph.2026.1896689

**Published:** 2026-08-26

**Authors:** Fei Tian, Tingting Li, Jiayu Duan, Jia Li, Guangpu Li, Dongwei Liu

**Affiliations:** 1 Research Institute of Nephrology, Zhengzhou University, Zhengzhou, China; 2 Henan Province Research Center For Kidney Disease, Zhengzhou, China; 3 Key Laboratory of Precision Diagnosis and Treatment for Chronic Kidney Disease in Henan Province, Zhengzhou, China; 4 Department of Integrated Traditional and Western Nephrology, the First Affiliated Hospital of Zhengzhou University, Zhengzhou, China

**Keywords:** cross-sectional study, NlpR, peritoneal dialysis, residual renal function, systemic inflammation

## Abstract

**Background:**

To investigate the association between the neutrophil-to-lymphocyte-platelet ratio (NLPR) and residual renal function (RRF) in early-stage peritoneal dialysis (PD) patients, and to evaluate its association with for RRF decline.

**Methods:**

This retrospective study included PD patients treated between January 2025 and February 2026. Patients were divided into an early group (dialysis duration 3–12 months) and a late group (>12 months). One extreme NLPR outlier was excluded from the early group, resulting in 23 patients in the early group and 49 in the late group. Spearman’s correlation, linear regression, and ROC curve analysis were used.

**Results:**

In the early group, NLPR was significantly negatively correlated with residual renal Ccr (*r* = -0.559), residual renal Kt/V (*r* = -0.465), eGFR (*r* = -0.471), nPCR (*r* = -0.515), and target Kt/V achievement (*r* = -0.448) (all *p* < 0.05). Univariate linear regression showed that NLPR significantly predicted residual renal Ccr (B = -1.502, *p* = 0.006), and it remained independently predictive after adjusting for nPCR (B = -1.592, *p* = 0.011). ROC curve analysis demonstrated good discriminatory ability for predicting lower RRF, with an AUC of 0.818 (95% CI 0.640-0.996, *p* = 0.010). The optimal cut-off was 10.372, with 81.8% sensitivity and 58.3% specificity. Subgroup analysis showed a stronger association in males (r = -0.796, *p* = 0.006).

**Conclusions:**

NLPR is significantly associated with RRF in early-stage PD patients. Although it demonstrated good discriminatory ability for RRF status in this cross-sectional study, its predictive value for RRF decline warrants further validation in longitudinal cohort studies.

## 1 Introduction

Peritoneal dialysis is one of the major renal replacement therapies for end-stage renal disease, offering advantages such as preservation of residual renal function, hemodynamic stability, and improved quality of life (1). Residual renal function is a key determinant of prognosis in PD patients, closely associated with volume status, nutritional status, inflammatory levels, and survival (2). Therefore, early identification of patients at high risk of residual renal function decline is of great importance for optimizing PD management and improving patient outcomes.

Systemic inflammation is common in PD patients and is strongly linked to adverse outcomes, including cardiovascular events, protein-energy wasting, and technique failure (3–7). In recent years, various novel inflammatory markers have been shown to have prognostic value in areas such as oncology and cardiovascular disease, due to their simplicity, low cost, and good reproducibility (8–10). Among these, simple ratios such as the neutrophil-to-lymphocyte ratio (NLR) and platelet-to-lymphocyte ratio (PLR) have been studied in PD patients (11–13). However, these markers only integrate two cellular components and may not fully capture the complexity of the inflammatory network.

More recently, composite inflammatory markers integrating neutrophils, lymphocytes, and platelets have gained attention. One such marker, the neutrophil-to-lymphocyte-platelet ratio (NLPR), has been reported to be associated with prognosis in patients with cardiovascular surgery-associated acute kidney injury and COVID-19 (14–16). This marker incorporates neutrophils (reflecting acute inflammation), lymphocytes (reflecting immune and nutritional status), and platelets (reflecting inflammation-related prothrombotic states), and may therefore provide a more comprehensive assessment of systemic inflammation than simple ratios. However, the relationship between NLPR and residual renal function in patients undergoing PD has not yet been reported.

Given this background, the present study aims to investigate the association between NLPR and residual renal function and dialysis adequacy in early-stage PD patients, to evaluate its association with for residual renal function status, and to preliminarily explore factors such as sex that might modify this association, with the goal of providing a simple and accessible reference marker for the early identification of patients at risk of residual renal function decline.

## 2 Materials and methods

### 2.1 Study population

This single-center retrospective study enrolled patients who underwent PD at the Department of Integrated Traditional Chinese and Western Medicine Nephrology, The First Affiliated Hospital of Zhengzhou University, from January 2025 to February 2026. The inclusion criterion was: completion of PD adequacy assessment (including complete blood count, biochemical tests, Kt/V, and Ccr) during follow-up. The exclusion criteria were: (1) dialysis duration < 3 months; (2) age < 18 years; (3) active infection or peritonitis within the previous 3 months; (4) comorbid malignancy, hepatitis B, hepatitis C, or coronary heart disease; (5) eGFR > 15 mL/min/1.73m².

For patients with multiple follow-up visits, the visit with peritoneal equilibration test (PET) data was preferentially selected; if PET data were available for multiple visits or for none, the earliest visit was selected. Extreme outliers were identified using boxplots (defined as data points exceeding three times the interquartile range). In the early group, one patient with an extreme NLPR value (72.122) was identified and excluded from the primary analysis. The final analysis included 23 patients in the early group. The core conclusions remained consistent before and after exclusion, indicating the robustness of the results.

This study was approved by the Ethical Review Committee for Research Projects of the First Affiliated Hospital of Zhengzhou University (approval number: 2026-KY-0787-001). The study was conducted in accordance with the principles of the Declaration of Helsinki. Informed consent was waived due to the retrospective design of the study.

### 2.2 Data collection

Demographic characteristics (age, sex), clinical data (dialysis duration, 24-hour urine volume, primary kidney disease, comorbidities), and laboratory findings were collected from the electronic medical record system. Laboratory parameters included complete blood count (neutrophils, lymphocytes, monocytes, platelets) and serum biochemistry (albumin, creatinine, blood urea nitrogen, C-reactive protein), all measured during the same follow-up period.

### 2.3 Calculation of inflammatory markers

The following inflammatory markers were calculated based on complete blood count and biochemistry results. The systemic inflammation response index (SIRI) was calculated as (neutrophils × monocytes)/lymphocytes. NLR was calculated as neutrophils/lymphocytes. PLR was calculated as platelets/lymphocytes (17). The monocyte-to-lymphocyte ratio (MLR) was calculated as monocytes/lymphocytes (18). NLPR was calculated as neutrophils/(lymphocytes × platelets) × 1000 (15). The C-reactive protein-to-albumin ratio (CAR) was calculated as C-reactive protein/albumin (19). The prognostic nutritional index (PNI) was calculated as albumin + 5 × lymphocytes (20).

### 2.4 Peritoneal dialysis-related parameters

Weekly Kt/V and weekly creatinine clearance (Ccr) were calculated according to the methods recommended by the K/DOQI guidelines (21). Residual renal Kt/V (rKt/V), residual renal Ccr (rCcr), peritoneal Kt/V, and peritoneal Ccr were calculated based on residual renal and dialysate urea and creatinine clearances. The normalized protein catabolic rate (nPCR) was calculated based on urea generation rate and body surface area (22). Adequate dialysis was defined as total Kt/V ≥ 1.7 or total Ccr ≥ 50 L/week/1.73m².

The peritoneal equilibration test (PET) was performed using a standard 4-hour dwell to record the dialysate-to-plasma creatinine ratio (D/Pcr). Because PET data were available only for a subset of patients, they were not included in the primary analysis, and the corresponding results are provided in the **Supplementary Material**.

### 2.5 Statistical analysis

SPSS version 24.0 was used for statistical analysis. Normality was tested using the Shapiro-Wilk test. Normally distributed continuous variables were expressed as mean ± standard deviation and compared between groups using the independent samples t-test. Non-normally distributed continuous variables were expressed as median (interquartile range) and compared using the Mann-Whitney U test. Categorical variables were expressed as frequency (percentage) and compared using the chi-square test or Fisher’s exact test.

Spearman’s rank correlation was used to analyze the associations between inflammatory markers and continuous PD parameters (e.g., rKt/V, eGFR). For binary outcomes (e.g., achievement of target Kt/V), the Mann-Whitney U test was used to compare inflammatory marker levels between groups.

Univariate linear regression analyses were performed with rCcr as the dependent variable and NLPR, NLR, or PLR as the independent variable, respectively, to compare the model fit (*R*²) among the three markers. To further evaluate the predictive performance of each marker for residual renal function status, ROC curves for NLPR, NLR, and PLR were generated, and the area under the curve (AUC), 95% confidence interval, and P value were calculated. Based on the NLPR ROC curve, the optimal cut-off value, sensitivity, and specificity for predicting lower residual renal function (dichotomized at the median rCcr value) were determined. Based on the above comparisons, linear regression was further performed with rCcr as the dependent variable and NLPR as the independent variable, and a multivariate model was constructed by adjusting for nPCR. Due to the limited sample size (*n* = 23), multivariate regression was performed using the Enter method. Covariates were selected based on clinical relevance and prior literature, rather than solely on univariate P values. nPCR was included as a key covariate because of its established role in reflecting both inflammatory and nutritional status in peritoneal dialysis patients, and its significant correlation with NLPR in univariate analysis (*r* = -0.515, *p* = 0.012). Model fit was assessed using adjusted *R*², and collinearity was evaluated using variance inflation factor (VIF). To assess the potential confounding effect of age, we performed a sensitivity analysis by additionally including age as a covariate in the multivariate regression model. Subgroup analyses were stratified by sex, median urine volume, and median dialysis duration. A two-sided *p* value < 0.05 was considered statistically significant.

## 3 Results

### 3.1 Patient selection and grouping

**Figure 1:**
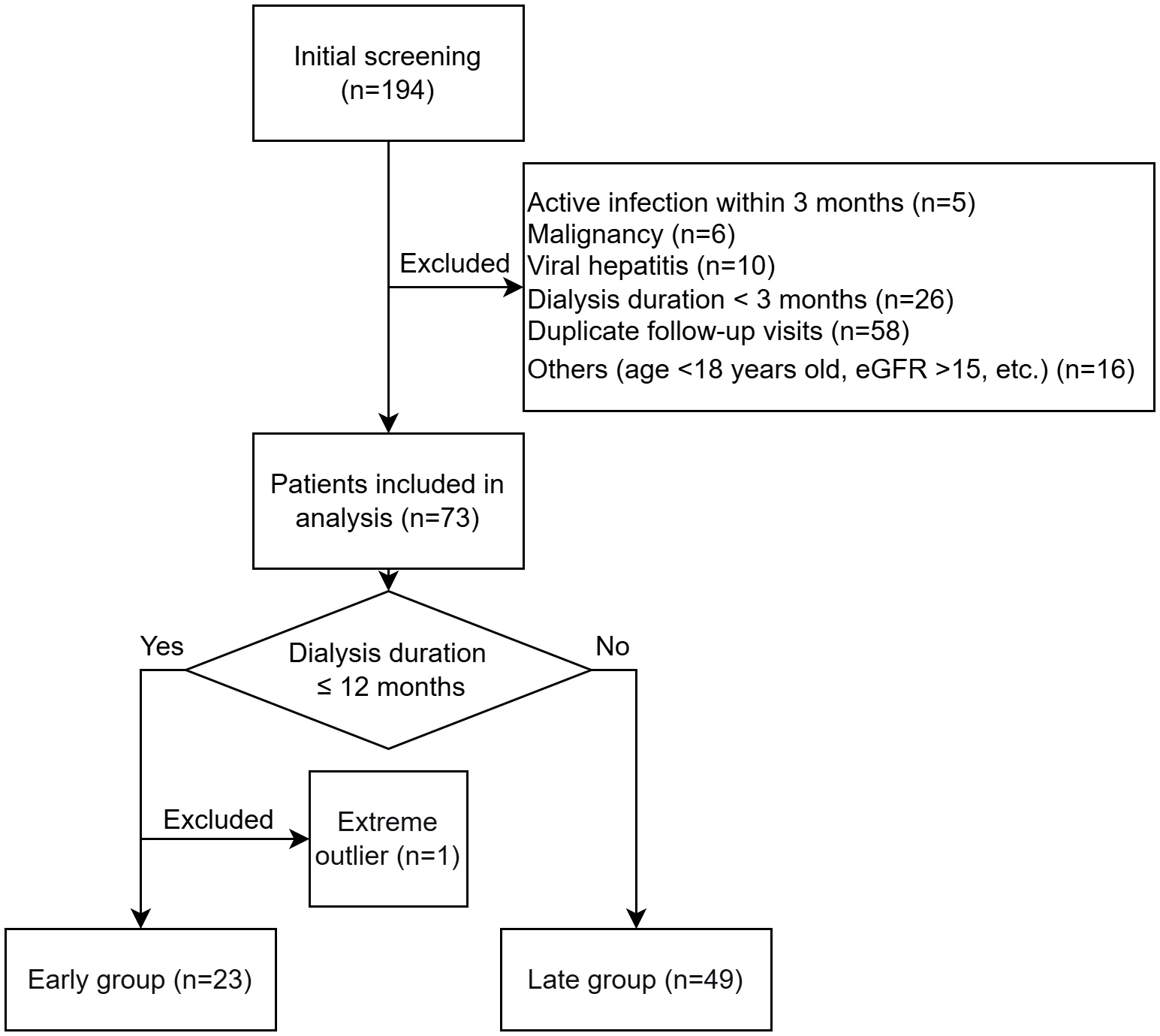
Flow chart of patient selection.

From January 1, 2025, to February 28, 2026, a total of 73 patients met the inclusion and exclusion criteria and were enrolled (**Figure 1**). Based on boxplot identification of outliers, one extreme outlier was excluded from the early group, resulting in a final analytic cohort of 23 patients in the early group and 49 patients in the late group. Baseline characteristics before outlier exclusion are presented in **Supplementary Table 1**.

### 3.2 Baseline characteristics

Baseline characteristics of the early and late groups are shown in **Table 1**. There were significant differences between the two groups in age, total Kt/V, rKt/V, rCcr, nPCR, and eGFR (all *p* < 0.05). The difference in 24-hour urine volume was of borderline significance (*p* = 0.058). No significant differences were observed in sex, hemoglobin, albumin, Kt/V target achievement rate, Ccr target achievement rate, or total Ccr (all *p* > 0.05). Comparisons of systemic inflammatory markers (SIRI, NLR, PLR, MLR, NLPR, CAR, PNI) between the two groups are presented in **Supplementary Table 2**, with only NLR showing a significant difference between the groups (*p* = 0.043).

**Table 1:** Baseline characteristics of patients in the early and late groups.

Variable	Early group (*n* = 23)	Late group (*n* = 49)	*p* value
Age (years)	37.13 ± 12.04	43.82 ± 12.62	0.037
Male, n(%)	10(43.5)	22(44.9)	0.910
Hemoglobin (g/L)	105.00(98.00,110.00)	105.00(90.00,117.50)	0.928
Albumin (g/L)	33.47 ± 4.91	34.29 ± 3.73	0.431
24-hour urine volume (mL)	810.0(500.0,1200.0)	350.0(0.0,1025.0)	0.058
eGFR (ml/min/1.73m^2^)	1.82(0.66,2.26)	0.65(0.00,2.16)	0.044
Dialysis duration (months)	5.3(4.3,7.8)	24.5(20.2,37.3)	<0.001
Achieved target Kt/V, n(%)	17(73.9)	29(59.2)	0.225
Achieved target Ccr, n(%)	15(65.2)	25(51.0)	0.258
Total Kt/V (/week)	1.99 ± 0.44	1.78 ± 0.35	0.037
Total Ccr (L/week/1.73m^2^)	59.49(46.59,71.72)	50.17(44.08,63.73)	0.107
Residual renal Kt/V (/week)	0.57(0.25,0.73)	0.16(0.00,0.55)	0.017
Residual renal Ccr (L/week/1.73m^2^)	22.88(10.08,31.92)	7.75(0.00,27.01)	0.048
nPCR (g/kg/day)	1.44(1.23,1.64)	1.26(1.12,1.46)	0.050

eGFR, estimated glomerular filtration rate; nPCR, normalized protein catabolic rate. Continuous variables with normal distribution are presented as mean ± standard deviation and were compared using the independent-samples t-test; those with non-normal distribution are presented as median (interquartile range) and were compared using the Mann-Whitney U test. Categorical variables are presented as frequency (percentage) and were compared using the chi-square test or Fisher's exact test.

### 3.3 Association between inflammatory markers and PD parameters

**Figure 2:**
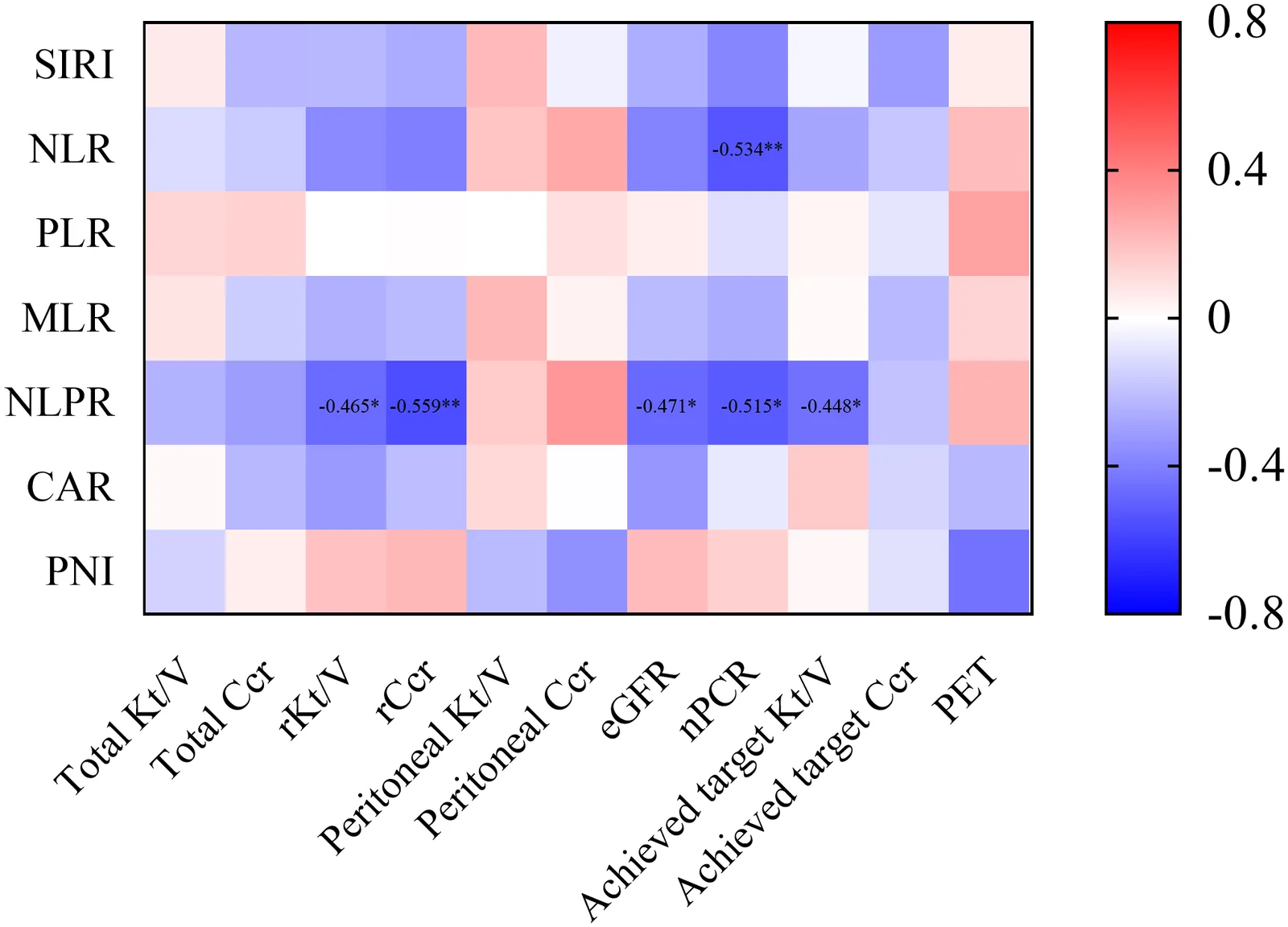
Heatmap of correlations between PD parameters and systemic inflammatory markers. The values in the cells are Spearman’s correlation coefficients (*r*). * indicates *p* < 0.05, ** indicates *p* < 0.01. Red represents positive correlation, blue represents negative correlation, and color intensity indicates the strength of the correlation. NLPR, neutrophil-to-lymphocyte-platelet ratio; NLR, neutrophil-to-lymphocyte ratio; PLR, platelet-to-lymphocyte ratio; MLR, monocyte-to-lymphocyte ratio; SIRI, systemic inflammation response index; CAR, C-reactive protein-to-albumin ratio; PNI, prognostic nutritional index; rKt/V, residual renal Kt/V; rCcr, residual renal Ccr; eGFR, estimated glomerular filtration rate; nPCR, normalized protein catabolic rate; PET, peritoneal equilibration test.

Associations between inflammatory markers and PD parameters in the early group (*n* = 23) are shown in the heatmap (**Figure 2**). NLR was significantly negatively correlated with nPCR (*r* = -0.534, *p* < 0.05). NLPR was significantly negatively correlated with rKt/V (*r* = -0.465), rCcr (*r* = -0.559), eGFR (*r* = -0.471), nPCR (*r* = -0.515), and achievement of target Kt/V (*r* = -0.448) (all *p* < 0.05), with the strongest correlation observed for rCcr. No other inflammatory markers (SIRI, PLR, MLR, CAR, PNI) showed significant correlations with any PD parameters (all *p* > 0.05). Complete correlation results for both groups are presented in **Supplementary Table 3** and **S4**.

### 3.4 Linear regression analysis of NLPR, NLR, and PLR with rCcr

**Figure 3:**
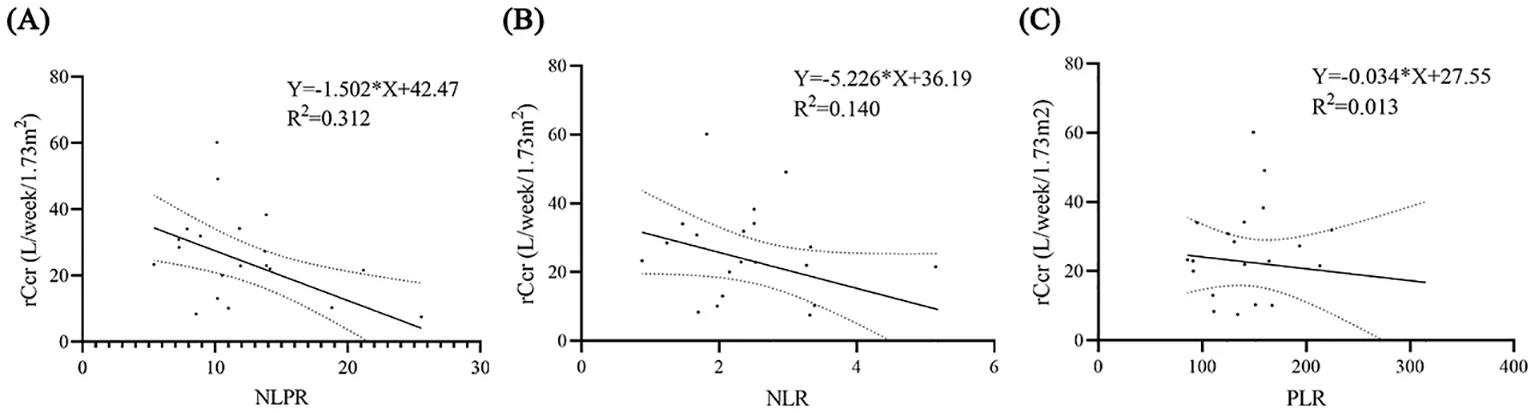
Univariate linear regression scatter plots of NLPR, NLR, and PLR with residual renal Ccr (early group). **(A)** NLPR, **(B)** NLR, **(C)** PLR. Solid lines represent linear regression fits, and dashed lines represent 95% confidence intervals. The regression equation for NLPR was Y = 42.47 - 1.502X, *R*² = 0.312, *p* = 0.006; for NLR, Y = 45.51 - 5.226X, *R*² = 0.140, *p* = 0.078; and for PLR, Y = 41.47 - 0.034X, *R*² = 0.013, *p* = 0.602. NLPR, neutrophil-to-lymphocyte-platelet ratio (neutrophils/(lymphocytes × platelets) × 1000); NLR, neutrophil-to-lymphocyte ratio; PLR, platelet-to-lymphocyte ratio; rCcr, residual renal creatinine clearance (L/week/1.73m²). In the regression equations, Y represents rCcr (L/week/1.73m²).

Univariate linear regression analyses were performed with rCcr as the dependent variable and NLPR, NLR, or PLR as the independent variable, respectively (**Figure 3**). The regression equation for NLPR was Y = 42.47 - 1.502X (*R*² = 0.312, *p* = 0.006); for NLR, Y = 45.51 - 5.226X (*R*² = 0.140, *p* = 0.078); and for PLR, Y = 41.47 - 0.034X (*R*² = 0.013, *p* = 0.602). NLPR showed the strongest explanatory power for rCcr (*R*² = 0.312), followed by NLR (*R*² = 0.140), and PLR (*R*² = 0.013).

Given that NLPR showed the best performance among the three markers in the above comparisons, multivariate linear regression was further performed with rCcr as the dependent variable, NLPR as the independent variable, and nPCR as a covariate. After adjusting for nPCR, NLPR remained significantly negatively associated with rCcr (B = -1.592, 95% CI -2.774 to -0.410, *p* = 0.011), with an adjusted *R*² of 0.247 for the model (**Table 2**). nPCR itself was not significantly associated with rCcr (*p* = 0.743). Collinearity diagnostics showed no significant collinearity between the independent variables (VIF = 1.296).

**Table 2:** Linear regression analysis of NLPR and residual renal Ccr (*n* = 23).

Variable	Univariate model		Multivariate model	
	B (95% CI)	*p* value	B (95% CI)	*p* value
NLPR	-1.502(-2.514 to -0.489)	0.006	-1.592(-2.774 to -0.410)	0.011
nPCR	–	–	-3.629(-26.389 to 19.131)	0.743
Adjusted *R*^2^	0.279		0.247	

The dependent variable is residual renal Ccr (L/week/1.73m²). The univariate model included only NLPR as an independent variable, while the multivariate model included both NLPR and nPCR.

### 3.5 ROC curve analysis of NLPR, NLR, and PLR for predicting residual renal function status

**Figure 4:**
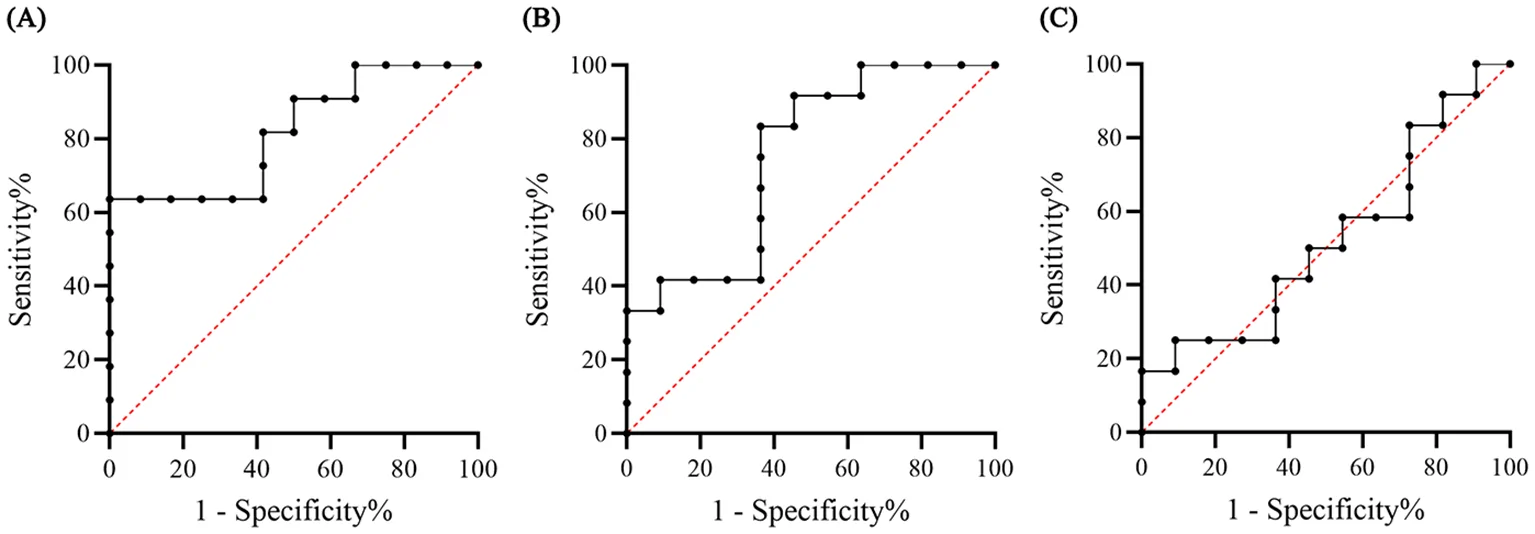
ROC curves of NLPR, NLR, and PLR for predicting lower residual renal function (early group). **(A)** NLPR, **(B)** NLR, **(C)** PLR. Using the median rCcr (22.88 L/week/1.73m²) as the cut-off, patients were divided into higher and lower residual renal function groups. NLPR showed an AUC of 0.818 (95% CI 0.640 to 0.996), *p* = 0.010; NLR showed an AUC of 0.750 (95% CI 0.545 to 0.955), *p* = 0.042; and PLR showed an AUC of 0.523 (95% CI 0.279 to 0.766), *p* = 0.854. The diagonal line represents the reference line (AUC = 0.5). NLPR: neutrophil-to-lymphocyte-platelet ratio (neutrophils/(lymphocytes × platelets) × 1000); NLR, neutrophil-to-lymphocyte ratio; PLR, platelet-to-lymphocyte ratio; AUC, area under the curve; rCcr, residual renal creatinine clearance (L/week/1.73m²).

Using the median residual renal Ccr (22.88 L/week/1.73m²) as the cut-off, patients in the early group were divided into higher and lower residual renal function groups. ROC curves for NLPR, NLR, and PLR were generated (**Figure 4**). NLPR showed an AUC of 0.818 (95% CI 0.640-0.996, *p* = 0.010), NLR showed an AUC of 0.750 (95% CI 0.545-0.955, *p* = 0.042), and PLR showed an AUC of 0.523 (95% CI 0.279-0.766, *p* = 0.854). These results indicate that NLPR had the best discriminatory ability for residual renal function status, followed by NLR, while PLR showed no significant predictive value. The maximum Youden index of the NLPR was 0.401, corresponding to an optimal cut-off value of 10.372, which yielded a sensitivity of 81.8% and a specificity of 58.3%.

### 3.6 Exploratory and sensitivity analyses

Exploratory and sensitivity analyses were performed to further assess the stability of the association between NLPR and residual renal function across different subgroups. Detailed results are presented in **Supplementary Tables 5**–**S7**.

Subgroup analysis showed that the negative correlation between NLPR and rCcr was more pronounced in male patients (*r* = -0.796, *p* = 0.006), while in female patients, the correlation was also negative but not statistically significant (*r* = -0.495, *p* = 0.086). When stratified by urine volume, both the low and high urine volume groups showed negative correlations, but neither reached statistical significance.

Sensitivity analysis showed that before outlier exclusion, the correlation coefficient between NLPR and rCcr was -0.608 (*p* = 0.002); after exclusion, it was -0.559 (*p* = 0.006). The effect sizes were similar and the direction of the association was consistent. In a further sensitivity analysis, when age was used as a covariate instead of nPCR in the multivariate regression model, NLPR remained significantly negatively associated with rCcr (B = -1.596, 95% CI -2.635 to -0.502, *p* = 0.006), with an adjusted *R*² of 0.253, consistent with the primary analysis.

## 4 Discussion

This study demonstrated that in early-stage PD patients, the novel inflammatory marker NLPR is significantly negatively correlated with residual renal function (rKt/V, rCcr, eGFR), dialysis adequacy (total Kt/V, achievement of target Kt/V), and nutritional status (nPCR). Univariate linear regression showed that NLPR was a significant predictor of rCcr (B = -1.502, *p* = 0.006), and it remained independently predictive after adjusting for nPCR (B = -1.592, *p* = 0.011). ROC curve analysis showed that NLPR had good discriminatory ability for predicting lower residual renal function (AUC = 0.818, *p* = 0.010), with an optimal cut-off value of 10.372. Subgroup analysis revealed that this association was more pronounced in male patients (*r* = -0.796, *p* = 0.006). These findings suggest that NLPR may be a potential inflammatory marker associated with residual renal function in early-stage PD patients; however, given the cross-sectional design of this study, causal inference and predictive value cannot be established.

NLPR integrates information from neutrophils, lymphocytes, and platelets, potentially reflecting systemic inflammatory status from multiple dimensions. Neutrophils are key effector cells of the inflammatory response; their increase suggests inflammation activation. Lymphocyte decrease reflects immune exhaustion or malnutrition. Platelet activation is associated with inflammation-related prothrombotic states (23–25). The association between NLPR and residual renal function decline may be explained by several mechanisms. First, systemic inflammation can directly damage residual kidneys via inflammatory cytokines (e.g., IL-6, TNF-α), accelerating glomerulosclerosis and interstitial fibrosis (26). Second, in this study, NLPR was significantly negatively correlated with nPCR (*r* = -0.515, *p* < 0.05), suggesting a close relationship between inflammation and protein-energy wasting. Malnutrition can further impair immune function and exacerbate inflammation, creating a vicious cycle that accelerates residual renal function loss (27). Third, increased platelet activation in inflammatory states may lead to renal microvascular endothelial dysfunction, affecting residual renal blood flow (28).

As a composite inflammatory marker integrating neutrophils, lymphocytes, and platelets, NLPR has recently been reported to be associated with kidney injury and patient prognosis in several clinical fields (14–16). For example, perioperative changes in NLPR effectively predict postoperative acute kidney injury in patients undergoing cardiovascular surgery (15). In the field of PD, previous studies have mostly focused on simple ratios such as NLR and PLR (13, 29, 30). For instance, Chen et al. reported that a high PLR can predict the risk of cardiovascular events in CAPD patients (13). In comparison, NLPR integrates more inflammatory pathway information and may therefore have higher sensitivity. In the present study, the correlation coefficient between NLPR and rCcr reached a moderate-to-strong level (*r* = -0.559), and the adjusted R² from the univariate regression was 0.279, indicating that NLPR explained 27.9% of the variance in rCcr. This effect size is acceptable for a single biomarker.

ROC curve analysis demonstrated that NLPR had an AUC of 0.818 for discriminating lower residual renal function, with an optimal cut-off of 10.372. This level of discrimination is considered good (0.8-0.9). It is noteworthy that residual renal function is influenced by multiple factors, including primary kidney disease, dialysis duration, nutritional status, and blood pressure control, making it unlikely that any single biomarker would achieve perfect predictive performance. Previous studies have shown that even in larger PD cohorts, the AUCs of single inflammatory markers for predicting patient outcomes are mostly in the range of 0.6-0.8. For example, Yang et al. reported an AUC of 0.644 for SII in predicting all-cause mortality in 369 PD patients (31). The performance of NLPR in the present study is comparable to these reports, suggesting its potential clinical value. In practice, NLPR could be used in combination with other clinical indicators (e.g., urine volume, nutritional status) to further improve predictive accuracy.

Subgroup analysis showed that the negative correlation between NLPR and rCcr was more pronounced in male patients (*r* = -0.796, *p* = 0.006), whereas in female patients, the correlation was also negative but did not reach statistical significance (*r* = -0.495, *p* = 0.086). This difference may be related to sex-specific metabolic characteristics. Male patients typically have higher muscle mass, and the association between muscle mass and clinical outcomes exhibits significant sex differences (32); thus, changes in residual renal function in males may be more sensitive to inflammatory status. Furthermore, estrogen has been shown to protect against kidney injury by inhibiting the TGF-βRI-SMAD pathway (33), which may partially offset the detrimental effects of inflammation on residual kidneys in female patients. Sex has been established as an important effect modifier of dialysis outcomes in the PD population (34). Given the relatively small sample size of female patients in this study (n=13), this finding requires validation in larger cohorts. Nevertheless, this observation suggests that future studies should consider sex as a potential effect modifier.

This study has several limitations. First, the sample size of the early group was relatively small (n=23), which limited the number of covariates that could be included in the multivariate regression model. We adjusted for nPCR as a key marker reflecting nutritional-inflammatory status, but other potential confounders—such as age, diabetes, PD vintage, and peritonitis history—were not included in the model due to sample size constraints. Notably, peritonitis history had no variability in this early-stage cohort (zero occurrence), precluding its inclusion in regression analysis. Therefore, our findings should be interpreted with caution, and larger prospective studies are warranted to confirm these results. Second, this was a single-center retrospective cross-sectional study, which precludes causal inference regarding the association between NLPR and residual renal function decline. In our cohort, only 9 of the 23 early-stage patients had repeated measurements, which was insufficient to support a reliable longitudinal analysis. Future prospective longitudinal studies with larger sample sizes are warranted to validate the predictive value of NLPR. Additionally, the single-center design may limit the generalizability of our findings. Third, PET data were only available for a subset of patients, and we could not systematically assess the impact of peritoneal transport characteristics on the results. Fourth, due to sample size constraints, we could not adjust for all potential confounding factors (e.g., medication use, primary kidney disease, specific dialysis prescriptions). Fifth, we excluded one extreme NLPR outlier (72.122); although sensitivity analyses showed similar effect sizes (-0.608 vs. -0.559) and consistent directions before and after exclusion, this may still have had some impact on the results. Finally, this is an exploratory analysis, and the conclusions need to be validated in larger, multicenter, prospective cohorts.

## 5 Conclusion

In conclusion, in early-stage PD patients, NLPR is significantly associated with residual renal function, dialysis adequacy, and nutritional status, and demonstrates good discriminatory ability for residual renal function status. However, due to the cross-sectional nature of this study, these findings should be interpreted as associations rather than predictions. Future prospective longitudinal studies are needed to determine whether NLPR can predict residual renal function decline.

## Data Availability

The original contributions presented in the study are included in the article/**Supplementary Material**. Further inquiries can be directed to the corresponding author/s.
