# Supplementary material for: Association of neutrophil-to-lymphocyte-platelet ratio with residual renal function in early-stage peritoneal dialysis patients
Source: Front Nephrol. 2026 Aug 26;6:1896689. doi: 10.3389/fneph.2026.1896689 (PMC13553526; doi:10.3389/fneph.2026.1896689)
Supplement: Supplementary file 1 [file Table1.docx]

| Table S1. Comparison of baseline characteristics between early and late groups before outlier exclusion | | | |
| --- | --- | --- | --- |
| Variable | Early group (*n*=24) | Late group (*n*=49) | *p* value |
| Age (years) | 37.5±11.91 | 43.82±12.62 | 0.045 |
| Male, n(%) | 11(45.8) | 22(44.9) | 0.940 |
| Hemoglobin (g/L) | 104.00(98.00,109.75) | 105.00(90.00,117.50) | 0.953 |
| Albumin (g/L) | 33.54±4.82 | 34.29±3.73 | 0.466 |
| 24-hour urine volume (mL) | 805.0(327.5,1175.0) | 350.0(0.0,1025.0) | 0.101 |
| eGFR (ml/min/1.73m^2^) | 1.81(0.65,2.24) | 0.65(0.00,2.16) | 0.080 |
| Dialysis duration (months) | 5.6(4.4,8.0) | 24.5(20.2,37.3) | <0.001 |
| Achieved target Kt/V, n(%) | 17(70.8) | 29(59.2) | 0.333 |
| Achieved target Ccr, n(%) | 15(62.5) | 25(51.0) | 0.355 |
| Total Kt/V (/week) | 1.96±0.45 | 1.78±0.35 | 0.065 |
| Total Ccr (L/week/1.73m^2^) | 59.33(45.95,71.29) | 50.17(44.08,63.73) | 0.188 |
| Residual renal Kt/V (/week) | 0.57(0.18,0.72) | 0.16(0.00,0.55) | 0.035 |
| Residual renal Ccr (L/week/1.73m^2^) | 22.43(8.82,31.64) | 7.75(0.00,27.01) | 0.087 |
| nPCR (g/kg/day) | 1.45(1.23,1.63) | 1.26(1.12,1.46) | 0.040 |
| SIRI | 0.959(0.626,1.291) | 1.042(0.697,1.607) | 0.307 |
| NLR | 2.509(1.855,3.326) | 3.000(2.237,4.027) | 0.091 |
| PLR | 143.998(114.467,163.228) | 162.281(124.210,194.578) | 0.089 |
| MLR | 0.233(0.215,0.301) | 0.272(0.231,0.348) | 0.134 |
| NLPR | 11.880(9.205,18.592) | 15.653(10.723,20.249) | 0.133 |
| CAR* | 0.023(0.012,0.094) | 0.037(0.019,0.125) | 0.193 |
| PNI | 40.950(37.975,43.050) | 41.100(37.525,43.600) | 0.842 |

Notes: eGFR, estimated glomerular filtration rate; nPCR, normalized protein catabolic rate; SIRI, systemic inflammation response index; NLR, neutrophil-to-lymphocyte ratio; PLR, platelet-to-lymphocyte ratio; MLR, monocyte-to-lymphocyte ratio; NLPR, neutrophil-to-lymphocyte-platelet ratio (neutrophils/(lymphocytes × platelets) × 1000); CAR, C-reactive protein-to-albumin ratio; PNI, prognostic nutritional index. *CAR data were missing in 2 cases in the early group and 1 case in the late group. Continuous variables with normal distribution are presented as mean ± standard deviation and were compared using the independent-samples t-test; those with non-normal distribution are presented as median (interquartile range) and were compared using the Mann-Whitney U test. Categorical variables are presented as frequency (percentage) and were compared using the chi-square test or Fisher's exact test.
