# Supplementary material for: Association of neutrophil-to-lymphocyte-platelet ratio with residual renal function in early-stage peritoneal dialysis patients
Source: Front Nephrol. 2026 Aug 26;6:1896689. doi: 10.3389/fneph.2026.1896689 (PMC13553526; doi:10.3389/fneph.2026.1896689)
Supplement: Supplementary file 2 [file Table2.docx]

| Table S2. Comparison of systemic inflammatory markers between early and late groups | | | |
| --- | --- | --- | --- |
| Variable | Early group (*n*=23) | Late group (*n*=49) | *p* value |
| SIRI | 0.914(0.617,1.248) | 1.042(0.697,1.607) | 0.178 |
| NLR | 2.509(1.817,3.320) | 3.000(2.237,4.027) | 0.043 |
| PLR | 140.854(111.236,164.341) | 162.281(124.210,194.578) | 0.094 |
| MLR | 0.231(0.214,0.284) | 0.272(0.231,0.348) | 0.070 |
| NLPR | 11.839(8.890,17.925) | 15.653(10.723,20.249) | 0.066 |
| CAR* | 0.021(0.011,0.116) | 0.037(0.019,0.125) | 0.183 |
| PNI | 40.689±4.871 | 40.718±4.632 | 0.980 |

Notes: SIRI, systemic inflammation response index; NLR, neutrophil-to-lymphocyte ratio; PLR, platelet-to-lymphocyte ratio; MLR, monocyte-to-lymphocyte ratio; NLPR, neutrophil-to-lymphocyte-platelet ratio (neutrophils/(lymphocytes × platelets) × 1000); CAR, C-reactive protein-to-albumin ratio; PNI, prognostic nutritional index. *CAR data were missing in 2 cases in the early group and 1 case in the late group. Continuous variables with normal distribution are presented as mean ± standard deviation and were compared using the independent-samples t-test; those with non-normal distribution are presented as median (interquartile range) and were compared using the Mann-Whitney U test.
