# Supplementary material for: Association of neutrophil-to-lymphocyte-platelet ratio with residual renal function in early-stage peritoneal dialysis patients
Source: Front Nephrol. 2026 Aug 26;6:1896689. doi: 10.3389/fneph.2026.1896689 (PMC13553526; doi:10.3389/fneph.2026.1896689)
Supplement: Supplementary file 3 [file Table3.docx]

| Table S3. Correlations between systemic inflammatory markers and PD parameters in the early group (*n*=23) | | | | | | | | |
| --- | --- | --- | --- | --- | --- | --- | --- | --- |
| PD indicators | Statistic | SIRI | NLR | PLR | MLR | NLPR | CAR## | PNI |
| Total Kt/V | *r* | 0.064 | -0.116 | 0.126 | 0.080 | -0.242 | 0.019 | -0.137 |
|  | *P* | 0.771 | 0.599 | 0.565 | 0.717 | 0.266 | 0.933 | 0.532 |
| Total Ccr | *r* | -0.231 | -0.169 | 0.138 | -0.157 | -0.312 | -0.223 | 0.047 |
|  | *P* | 0.288 | 0.441 | 0.529 | 0.474 | 0.147 | 0.330 | 0.830 |
| Residual renal Kt/V | *r* | -0.225 | -0.369 | 0.000 | -0.248 | **-0.465** | -0.317 | 0.192 |
|  | *P* | 0.303 | 0.083 | 1.000 | 0.253 | **0.025** | 0.161 | 0.380 |
| Residual renal Ccr | *r* | -0.264 | -0.405 | 0.006 | -0.215 | **-0.559** | -0.209 | 0.224 |
|  | *P* | 0.223 | 0.056 | 0.979 | 0.325 | **0.006** | 0.363 | 0.305 |
| Peritoneal Kt/V | *r* | 0.218 | 0.184 | -0.002 | 0.222 | 0.162 | 0.117 | -0.212 |
|  | *P* | 0.317 | 0.401 | 0.993 | 0.308 | 0.460 | 0.614 | 0.330 |
| Peritoneal Ccr | *r* | -0.047 | 0.272 | 0.100 | 0.039 | 0.325 | -0.009 | -0.353 |
|  | *P* | 0.830 | 0.210 | 0.650 | 0.861 | 0.130 | 0.969 | 0.099 |
| eGFR | *r* | -0.257 | -0.391 | 0.050 | -0.215 | **-0.471** | -0.331 | 0.212 |
|  | *P* | 0.236 | 0.065 | 0.819 | 0.325 | **0.023** | 0.142 | 0.332 |
| nPCR | *r* | -0.382 | **-0.534** | -0.105 | -0.263 | **-0.515** | -0.070 | 0.151 |
|  | *P* | 0.072 | **0.009** | 0.634 | 0.226 | **0.012** | 0.763 | 0.491 |
| PET# | *r* | 0.060 | 0.210 | 0.294 | 0.133 | 0.235 | -0.224 | -0.445 |
|  | *P* | 0.854 | 0.512 | 0.353 | 0.680 | 0.463 | 0.484 | 0.147 |
| Achieved target Kt/V | *r* | -0.030 | -0.284 | 0.030 | 0.015 | **-0.448** | 0.166 | 0.030 |
|  | *P* | 0.892 | 0.190 | 0.892 | 0.946 | **0.032** | 0.472 | 0.892 |
| Achieved target Ccr | *r* | -0.317 | -0.179 | -0.083 | -0.220 | -0.193 | -0.133 | -0.096 |
|  | *P* | 0.141 | 0.414 | 0.708 | 0.313 | 0.378 | 0.564 | 0.662 |
| Notes: SIRI, systemic inflammation response index; NLR, neutrophil-to-lymphocyte ratio; PLR, platelet-to-lymphocyte ratio; MLR, monocyte-to-lymphocyte ratio; NLPR, neutrophil-to-lymphocyte-platelet ratio (neutrophils/(lymphocytes × platelets) × 1000); CAR, C-reactive protein-to-albumin ratio; PNI, prognostic nutritional index. PET, peritoneal equilibration test; eGFR, estimated glomerular filtration rate; nPCR, normalized protein catabolic rate. #PET data were available for 12 cases; ##CAR data were available for 21 cases; Data for the correlation between PET and CAR were available for 12 cases (cases with both PET and CAR data). Bold values indicate *p* < 0.05. | | | | | | | | |
