# Supplementary material for: Association of neutrophil-to-lymphocyte-platelet ratio with residual renal function in early-stage peritoneal dialysis patients
Source: Front Nephrol. 2026 Aug 26;6:1896689. doi: 10.3389/fneph.2026.1896689 (PMC13553526; doi:10.3389/fneph.2026.1896689)
Supplement: Supplementary file 4 [file Table4.docx]

| Table S4. Correlations between systemic inflammatory markers and PD parameters in the late group (*n*=49) | | | | | | | | |
| --- | --- | --- | --- | --- | --- | --- | --- | --- |
| PD indicators | Statistic | SIRI | NLR | PLR | MLR | NLPR | CAR## | PNI |
| Total Kt/V | *r* | -0.036 | 0.067 | 0.115 | -0.153 | -0.036 | 0.134 | 0.065 |
|  | *P* | 0.805 | 0.649 | 0.432 | 0.295 | 0.804 | 0.366 | 0.657 |
| Total Ccr | *r* | 0.073 | 0.097 | -0.252 | -0.021 | **0.322** | 0.119 | 0.003 |
|  | *P* | 0.617 | 0.505 | 0.081 | 0.888 | **0.024** | 0.421 | 0.986 |
| Residual renal Kt/V | *r* | 0.010 | 0.018 | -0.201 | -0.103 | 0.175 | 0.004 | -0.064 |
|  | *P* | 0.948 | 0.904 | 0.166 | 0.481 | 0.229 | 0.976 | 0.664 |
| Residual renal Ccr | *r* | 0.027 | 0.058 | -0.208 | -0.093 | 0.229 | -0.003 | -0.046 |
|  | *P* | 0.856 | 0.692 | 0.152 | 0.524 | 0.113 | 0.982 | 0.755 |
| Peritoneal Kt/V | *r* | -0.169 | -0.099 | 0.207 | -0.114 | -0.279 | -0.045 | 0.034 |
|  | *P* | 0.246 | 0.498 | 0.154 | 0.435 | 0.053 | 0.762 | 0.819 |
| Peritoneal Ccr | *r* | -0.008 | 0.005 | 0.075 | 0.107 | 0.013 | 0.134 | -0.032 |
|  | *P* | 0.958 | 0.974 | 0.608 | 0.466 | 0.932 | 0.366 | 0.829 |
| eGFR | *r* | 0.045 | 0.045 | -0.196 | -0.068 | 0.209 | 0.004 | -0.072 |
|  | *P* | 0.758 | 0.759 | 0.178 | 0.642 | 0.150 | 0.979 | 0.625 |
| nPCR | *r* | 0.057 | 0.103 | 0.003 | -0.084 | 0.046 | -0.033 | 0.220 |
|  | *P* | 0.698 | 0.481 | 0.985 | 0.568 | 0.755 | 0.824 | 0.128 |
| PET# | *r* | 0.165 | 0.004 | -0.001 | 0.284 | 0.135 | -0.057 | -0.261 |
|  | *P* | 0.383 | 0.981 | 0.995 | 0.129 | 0.477 | 0.767 | 0.164 |
| Achieved target Kt/V | *r* | -0.023 | 0.129 | 0.164 | -0.120 | 0.006 | 0.171 | 0.015 |
|  | *P* | 0.873 | 0.376 | 0.259 | 0.410 | 0.968 | 0.246 | 0.920 |
| Achieved target Ccr | *r* | 0.144 | 0.121 | -0.240 | 0.013 | 0.254 | 0.044 | 0.117 |
|  | *P* | 0.322 | 0.407 | 0.097 | 0.929 | 0.078 | 0.768 | 0.424 |
| Notes: SIRI, systemic inflammation response index; NLR, neutrophil-to-lymphocyte ratio; PLR, platelet-to-lymphocyte ratio; MLR, monocyte-to-lymphocyte ratio; NLPR, neutrophil-to-lymphocyte-platelet ratio (neutrophils/(lymphocytes × platelets) × 1000); CAR, C-reactive protein-to-albumin ratio; PNI, prognostic nutritional index. PET, peritoneal equilibration test; eGFR, estimated glomerular filtration rate; nPCR, normalized protein catabolic rate. #PET data were available for 30 cases; ##CAR data were available for 48 cases; Data for the correlation between PET and CAR were available for 29 cases (cases with both PET and CAR data). Bold values indicate *p* < 0.05. | | | | | | | | |
