# Supplementary material for: Association of neutrophil-to-lymphocyte-platelet ratio with residual renal function in early-stage peritoneal dialysis patients
Source: Front Nephrol. 2026 Aug 26;6:1896689. doi: 10.3389/fneph.2026.1896689 (PMC13553526; doi:10.3389/fneph.2026.1896689)
Supplement: Supplementary file 5 [file Table5.docx]

| Table S5. Subgroup analysis correlation between NLPR and residual renal Ccr | | | |
| --- | --- | --- | --- |
| Subgroup | *n* | *r* | *p* |
| Sex |  |  |  |
| —Male | 10 | -0.796 | 0.006 |
| —Female | 13 | -0.495 | 0.086 |
| Urine volume |  |  |  |
| —Low urine volume group | 11 | -0.532 | 0.092 |
| —High urine volume group | 12 | -0.350 | 0.256 |
| Note: The median urine volume was 810 mL/24h. Patients were divided into low urine volume group (< 810 mL) and high urine volume group (≥ 810 mL) based on this cut-off value. Spearman's correlation coefficient (*r*) represents the correlation between NLPR and residual renal Ccr. | | | |
