# Supplementary material for: Association of neutrophil-to-lymphocyte-platelet ratio with residual renal function in early-stage peritoneal dialysis patients
Source: Front Nephrol. 2026 Aug 26;6:1896689. doi: 10.3389/fneph.2026.1896689 (PMC13553526; doi:10.3389/fneph.2026.1896689)
Supplement: Supplementary file 6 [file Table6.docx]

| Table S6. Sensitivity analysis: comparison of correlations before and after outlier exclusion (NLPR vs residual renal Ccr in the early group) | | | |
| --- | --- | --- | --- |
| Analysis | *n* | *r* | *p* |
| Before outlier exclusion | 24 | -0.608 | 0.002 |
| After outlier exclusion | 23 | -0.559 | 0.006 |
| Note: The early group sample size was 24 before outlier exclusion (including the patient with NLPR = 72.122) and 23 after outlier exclusion. Spearman's correlation coefficient (*r*) represents the correlation between NLPR and residual renal Ccr. | | | |
