# Supplementary material for: Association of neutrophil-to-lymphocyte-platelet ratio with residual renal function in early-stage peritoneal dialysis patients
Source: Front Nephrol. 2026 Aug 26;6:1896689. doi: 10.3389/fneph.2026.1896689 (PMC13553526; doi:10.3389/fneph.2026.1896689)
Supplement: Supplementary file 7 [file Table7.docx]

| Table S7. Sensitivity analysis: covariates in multivariate regression | | | | |
| --- | --- | --- | --- | --- |
| Model | Independent variables | B (95% CI) | *p* | Adjusted *R*^2^ |
| Model 1 | NLPR + nPCR | -1.592（-2.774至-0.410） | 0.011 | 0.247 |
| Model 2 | NLPR + age | -1.596（-2.635至-0.502） | 0.006 | 0.253 |
| Note: Model 1 (primary analysis) was adjusted for nPCR, and Model 2 (sensitivity analysis) was adjusted for age. B represents the unstandardized regression coefficient, and 95% CI represents the confidence interval. The dependent variable was residual renal Ccr. | | | | |
